# Late-Onset Tay-Sachs Disease - expanding the clinical phenotype

**DOI:** 10.5334/tohm.750

**Published:** 2023-01-31

**Authors:** Stela Lefter, Aisling M. Ryan

**Affiliations:** 1 Department of Neurology, Beaumont hospital, Dublin, Ireland; 2 Department of Neurology, Cork University Hospital, Cork, Ireland

**Keywords:** Tay-Sachs disease, neuromuscular, belly-dancer’s dyskinesia

We read with great interest the article “Diagnostic Tips from a Video Series and Literature Review of Patients with Late-Onset Tay-Sachs Disease” [1] by Riboldi and Lau [1]. The authors characterised their own cohort of 9 patients with LOTS including a detailed clinical and video description of the most characteristic features. In addition, *via* a Pubmed literature review, the authors identified 76 LOTS patients presenting with a neuromuscular, cerebellar, psychiatric, stuttering, or movement disorder phenotype. The authors then summarized the predominant clinical phenotypes, highlighting the diagnostic clues to guide the diagnosis of LOTS, for neurology specialists (neuromuscular, movement disorders) and psychiatrists. Diagnostic tips included triceps sign, distinct speech patterns, early psychiatric presentation, as well as cerebellar and neuromuscular signs in patients with a prominent psychiatric presentation.

We would like to highlight our experience in a recently published case series of LOTS affecting 5 out of 10 siblings, demonstrating marked clinical heterogeneity, and associated with a known *HEXA* variant 1073 + 1G > A and a novel variant c.459 + 24G > C [2].

According to Riboldi and Lau findings, clinical manifestations seemed to be consistent among patients in the same family. However, this was not the case in our family, with significant intra-familial heterogeneity in terms of disease onset, severity, and clinical phenotype. Clinical manifestations in our family emerged in various combinations over the years, including neuromuscular, various extrapyramidal (axial and distal extremity dystonia, chorea, tremor), spinocerebellar, and neuropsychiatric involvement. We have also illustrated these with videos of 3 siblings. One sibling had a combination of diffuse muscle wasting without fasciculations, pyramidal and cerebellar signs, as well as a complex hyperkinetic movement disorder featuring limb dystonia, chorea, and abdominal wall dyskinesia “belly dancer’s dyskinesia” – the latter previously unreported in LOTS.

All affected siblings in our family had neuropsychiatric problems (psychosis, depression, bipolar disorders) of various degrees, this being the presenting feature in 2 cases. Mental retardation (mild-moderate) was present in all 5 siblings, of which 2 had seizures as well. A wide range of (mild-severe) cognitive impairment was found in all siblings, of which 1 patient had frontal predominant features coupled with marked frontal atrophy on MRI – previously unreported in LOTS.

All affected siblings progressed to develop cerebellar gait ataxia of varying severity, with 1 sibling at the extreme of the spectrum who lost ambulation in her early 30s. Severe cerebellar atrophy was found in 3 of our imaged patients.

We agree with Riboldi and Lau that adult patients with LOTS are often diagnosed late, and after several misdiagnoses. The diagnosis of LOTS should be added to the list of differential diagnosis of anterior horn cell disorders, neuromuscular diseases, cerebellar ataxia and in patients with cerebellar atrophy on MRI, as well as in new-onset psychosis or mood disorders even in the absence of neurological signs. A positive family history of these manifestations is always a valuable clue. Further reports regarding the natural evolution and clinical heterogeneity of this rare condition are welcome to guide us towards earlier diagnosis and facilitate patient participation in clinical trials.
